# The T1405N Carbamoyl Phosphate Synthetase Polymorphism Does Not Affect Plasma Arginine Concentrations in Preterm Infants

**DOI:** 10.1371/journal.pone.0010792

**Published:** 2010-05-25

**Authors:** Rob M. J. Moonen, Iballa Reyes, Giacomo Cavallaro, Gema González-Luis, Jaap A. Bakker, Eduardo Villamor

**Affiliations:** 1 Department of Pediatrics, School for Oncology and Developmental Biology (GROW), Maastricht University Medical Center (MUMC+), Maastricht, The Netherlands; 2 Department of Pediatrics, Atrium Medical Centre Parkstad, Heerlen, The Netherlands; 3 Department of Pediatrics, Hospital Universitario Materno-Infantil de Canarias, Las Palmas de Gran Canaria, Spain; 4 Institute of Pediatrics and Neonatology, Fondazione IRCCS Ospedale Maggiore Policlinico, Mangiagalli e Regina Elena, University of Milan, Milan, Italy; 5 Department of Clinical Genetics, Maastricht University Medical Center (MUMC+), Maastricht, The Netherlands; AgroParisTech, France

## Abstract

**Background:**

A C-to-A nucleotide transversion (T1405N) in the gene that encodes carbamoyl-phosphate synthetase 1 (CPS1) has been associated with changes in plasma concentrations of L-arginine in term and near term infants but not in adults. In preterm infants homozygosity for the *CPS1* Thr1405 variant (CC genotype) was associated with an increased risk of having necrotizing enterocolitis (NEC). Plasma L-arginine concentrations are decreased in preterm infants with NEC.

**Aim:**

To examine the putative association between the *CPS1* T1405N polymorphism and plasma arginine concentrations in preterm infants.

**Methods:**

Prospective multicenter cohort study. Plasma and DNA samples were collected from 128 preterm infants (<30 weeks) between 6 and 12 hours after birth. Plasma amino acid and *CPS1* T1405N polymorphism analysis were performed.

**Results:**

Distribution of genotypes did not differ between the preterm (CC∶CA∶AA = 55.5%∶33.6%∶10.9%, n = 128) and term infants (CC∶CA∶AA = 54.2%∶35.4%∶10.4%, n = 96). There was no association between the *CPS1* genotype and plasma L-arginine or L-citrulline concentration, or the ornithine to citrulline ratio, which varies inversely with CPS1 activity. Also the levels of asymmetric dimethylarginine, and symmetric dimethylarginine were not significantly different among the three genotypes.

**Conclusions:**

The present study in preterm infants did not confirm the earlier reported association between *CPS1* genotype and L-arginine levels in term infants.

## Introduction

Necrotizing enterocolitis (NEC) remains a leading cause of morbidity and mortality in neonatal intensive care units. Although several predisposing factors have been identified, the exact etiology of NEC is unknown. Suspected pathophysiological mechanisms include the developmental immaturity of gastrointestinal motility, digestive ability, circulatory regulation, intestinal barrier function, and immune defence [1]–[5]. In the last years, numerous research efforts focused on identifying biomarkers for infants at risk for NEC [6], [7].

Nitric oxide (NO) has attracted considerable attention in the pathophysiology of NEC as it pertains to the regulation of intestinal blood flow and plays a key role in the maintenance of mucosal integrity, intestinal barrier function, and post-injury intestinal reparation [8]–[12]. NO is generated by NO synthase (NOS) during the enzymatic conversion of L-arginine to L-citrulline. The methylated arginine metabolite asymmetric dimethylarginine (ADMA) competitively inhibits L-arginine uptake of endothelial cells and NOS activity [13], [14]. ADMA has emerged as an early marker and/or mediator of endothelial dysfunction and it has been proved to be a novel, independent risk factor of cardiovascular and metabolic diseases [15].

The NOS substrate L-arginine is an essential amino acid for young mammals [16], [17]. Metabolic and molecular studies indicate that the underdevelopment of intestinal arginine synthesis may be primarily responsible for remarkably low plasma arginine concentrations in preterm neonates [17], [18]. Several studies demonstrated that plasma arginine concentrations are even more decreased in premature infants with NEC [14], [17], [19]–[21]. Moreover, arginine supplementation reduced the incidence of NEC in one small randomized controlled study [19].

The first step arginine formation occurs inside the mitochondrion and is catalyzed by the enzyme, carbamyl-phosphate synthetase I (CPS1). A specific single nucleotide polymorphism designated as T1405N in the *CPS1* gene results in a threonine to asparagine amino acid substitution at an important cofactor binding site. Previous studies demonstrated the association of the *CPS1* T1405N genotype with clinical situations where endogenous NO production is critically important, such as neonatal pulmonary hypertension, increased pulmonary artery pressure following surgical repair of congenital heart defects, or hepatovenocclusive disease following bone marrow transplantation [18], [22], [23].

Recently, we observed in a retrospective case-control study that homozygosity for the *CPS1* Thr1405 variant (CC T1405N genotype) was associated with an increased risk of having NEC [24]. This corresponded with the observation that term newborns with the CC T1405N genotype had significantly lower levels of L-arginine and NO metabolites than infants with the AA T1405N genotype (homozygous for the Asn1405 variant) [18]. However this association between *CPS1* T1405N genotype and L-arginine levels was not present in adults [22] and has not been yet studied in preterm infants. In the present work, we hypothesized that the *CPS1* T1405N genotype would influence the plasmatic concentrations of L-arginine, L-citrulline, and methylated arginine metabolites in preterm infants shortly after birth.

## Methods

### Patients and study design

Between July 2007 and October 2008 we performed a prospective multicenter cohort study. All infants with a gestational age <30 weeks and birth weight <1500 g born in this period and admitted to the level III neonatal intensive care unit of the Maastricht University Medical Center (The Netherlands), Hospital Universitario Materno-Infantil de Canarias (Las Palmas de Gran Canaria, Spain), and Carlo Poma Hospital (Mantova, Italy) were eligible for participation in the study, which was approved the Local Research Ethics Committees of the participating centres and registered in ClinicalTrials.gov Protocol Registration System (NCT00554866). Written informed consent from the parents was obtained. Exclusion criteria were the following: blood transfusion, enteral or parenteral protein intake, or inhaled NO administration before blood sampling. Therefore, none of the included infants received enteral nutrition and parenteral nutrition consisted only of dextrose and electrolytes. In addition, none of the included infants received insulin treatment before or during sampling. One blood sample (500 µL) was obtained between 6 and 12 hours after birth from an umbilical-artery or peripheral artery catheter. When not available, the blood sample was obtained from venous puncture. Immediately after collection, heparinized blood samples were put on ice and centrifuged within 10 minutes (4000 rpm, 10 min, 4°C) to obtain plasma. The plasma was deproteinised with 6 mg of solid 5-sulphosalicylic acid (SSA, Sigma, St. Louis, MO) per 100 µL plasma, and stored at −80°C until further analysis. Buccal cell samples for DNA testing were obtained with a sterile OmniSwab (Whatman, Sanford, ME), collected in Eppendorf sterile PCR tubes, and stored at −80°C until further analysis. The samples obtained in Las Palmas and Mantova were transported on dry ice to Maastricht where all the analysis were performed. Data on clinical characteristics were retrieved from medical records. To provide some data about the genotype distribution of the general population in the Netherlands, Spain and Italy, bucall cell samples were obtained from a group of healthy term infants.

### Analysis of the T1405N polymorphism in the *CPS1* gene

DNA was extracted using standard methods and stored at −20°C until genotyping. A 214-bp fragment encompassing the 4332 C>A polymorphism in exon 36 of the *CPS1* gene was amplified using polymerase chain reaction (PCR). Primers used were (forward) GCM357 5′-TAAATGCAGCTGTTTG CCAC-3′ and (reverse) GCM358 5′-GACTTGCAATCAAGTAAGGTGAAA-3′. The PCR mix consisted of 1X GeneAmp PCR Buffer II (Perkin-Elmer, Branchburg, NJ), 0.2 mM deoxyribonucleoside triphosphate (Pharmacia Biotech, Bridgewater, NJ), 1.5 mM MgCl2 (Perkin-Elmer, Branchburg, NJ), 250 nM of both primers, and 0.025 U/µL of AmpliTaq Gold (Perkin-Elmer, Branchburg, NJ). Thermocycling conditions started with an initial denaturation of 10 min 95°C, followed by 35 cycles of 95°C (45 s), 55°C (45 s), 72°C (45 s), and ended with a final extension step of 10 min at 72°C. The PCR product was purified and directly sequenced using the reverse primer.

### Plasma amino acid analysis

Plasma amino acid concentrations were analyzed by high-performance liquid chromatography as previously described [25].

Dimethylargines, ADMA and SDMA, were determined in plasma using an ultra-performance liquid chromatography (UPLC) separation module coupled to an electrospray ionisation tandem mass spectrometry (ESI-MS/MS, Quattro Premier, Waters, Etten-Leur, The Netherlands). Separation of the components of interest was adapted form the described method for the determination of amino acids [26]. Briefly, plasma samples were mixed with stable isotope labelled ADMA and deproteinised with SSA and diluted. ADMA and SDMA were detected in the multiple reaction mode (MRM) in ESI positive mode [27].

### Statistics

Assumptions of sample size were based on on data provided by Pearson et al, Summar et al and Moonen et al [18], [22], [24]. Sample size was calculated to detect a difference of 10 µmol/L (SD 8) in mean L-arginine concentration between the AA and the CC genotypes. We found that we needed 11 subjects per group to detect this difference. Assuming a 10–12% incidence of the AA genotype [22], [24], 92–110 patients would be necessary to include the 11 patients with AA genotype.

To determine whether polymorphisms of the *CPS1* gene were in Hardy-Weinberg equilibrium, the frequencies of alleles and of genotypes were analyzed, and actual and predicted genotype frequencies were compared by χ^2^ analysis with one degree of freedom. Results for continuous variables are expressed as mean (SD) or, if variables were not normally distributed, as median (interquartile range). Differences between mean values were assessed by one-way ANOVA followed by Bonferroni's post hoc t-test, t-test or the Mann-Whitney U test, as appropriate. Differences were considered significant at a P<0.05. All analyses were performed using GraphPad Prism (version 5.00 for Windows, GraphPad Software, San Diego California USA).

## Results

In total 128 preterm infants were enrolled in the study. Of these 128 infants, 31 were enrolled in Maastricht, 66 in Las Palmas de Gran Canaria and 31 in Mantova. The clinical characteristics of the patients are summarized in Table 1. Bucall cell samples were obtained from 96 healthy term infants (25 in Maastricht, 31 in Las Palmas de Gran Canaria and 40 in Mantova).

**Table 1:** Baseline patient characteristics.

	Total study group (n = 128)	CC genotype (n = 71)	CA genotype (n = 43)	AA genotype (n = 14)	*p*
Antenatal corticosteroids	*101 (79)*	*58 (82)*	*31 (72)*	*12 (86)*	NS
C-section	*83 (65)*	*49 (69)*	*28 (65)*	*6 (43)*	NS
Gestational age (wks)	28.1 [27.3–29.6]	28.1 [27.4–29.6]	28.1 [27.2–29.1]	29.1 [26.4–29.7]	NS
Birth weight (g)	1037 (SD 241)	1038 (SD 250)	1028 (SD 229)	1059 (SD 252)	NS
Male sex	*76 (59)*	*41 (58)*	*28 (65)*	*7 (50)*	NS
Death before discharge	*12 (9)*	*9 (13)*	*3 (7)*	*0 (0)*	NS
CRIB score	2 [1]–[5]	2 [1]–[5]	2 [1]–[4]	2 [1]–[6]	NS

Results are expressed as mean (SD), median [interquartile range], or *absolute numbers of patients (percentage)*.

Statistical analyses between the different genotypes. NS = not significant.

The distribution of the *CPS1* genotypes for the polymorphism at position 4332 within the overall study population fulfilled Hardy-Weinberg criteria. Genotype distribution (CC denotes homozygosity for the C-encoded Thr1405 variant, AA homozygosity for the A-encoded Asn1405 variant and AC heterozygosity for this polymorphism at position 1405) in preterm infants (CC∶CA∶AA = 55.5%∶33.6%∶10.9%, n = 128) (Table 2) did not significantly differ from genotype distribution in term infants (CC∶CA∶AA = 54.2%∶35.4%∶10.4%, n = 96) (Table 3). No significant differences among the three centers were observed in genotype distribution in preterm (Table 2) or term (Table 3) infants. As shown in Table 1, there were no significant differences in the clinical characteristics among the three *CPS1* genotypes.

**Table 2:** Distribution of the *CPS1* genotypes for the polymorphism at position 1405 in preterm infants.

	CC genotype	CA genotype	AA genotype
Las Palmas de Gran Canaria (Spain), n = 66	39 (59.1)	19 (28.8)	8 (12.1)
Mantova (Italy), n = 31	18 (58.1)	12 (38.7)	1 (3.2)
Maastricht (the Netherlands), n = 31	14 (45.2)	12 (38.7)	5 (16.1)
Total study group, n = 128	71 (55.5)	43 (33.6)	14 (10.9)

Results are expressed as absolute numbers of patients (percentage). CC denotes homozygosity for the C-encoded Thr1405 variant, AA homozygosity for the A-encoded Asn1405 variant, and AC heterozygosity for this polymorphism.

**Table 3:** Distribution of the *CPS1* genotypes for the polymorphism at position 1405 in healthy term infants.

	CC genotype	CA genotype	AA genotype
Las Palmas de Gran Canaria (Spain), n = 31	19 (61.3)	7 (22.6)	5 (16.1)
Mantova (Italy), n = 40	22 (55.0)	15 (37.5)	3 (7.5)
Maastricht (the Netherlands, ref. 24), n = 25	11 (44.0)	12 (48.0)	2 (8.0)
Total study group, n = 96	52 (54.2)	34 (35.4)	10 (10.4)

Results are expressed as absolute numbers of patients (percentage). CC denotes homozygosity for the C-encoded Thr1405 variant, AA homozygosity for the A-encoded Asn1405 variant, and AC heterozygosity for this polymorphism.

As shown in table 4, when examining urea cycle intermediates in relation to the distribution of the *CPS1* genotypes, we found no significant differences in arginine or citrulline concentrations. The levels of ornithine and the ornithine:citrulline ratio were also not significantly different among the three genotypes. Concentrations of other amino acids in plasma also showed no significant differences among the three genotypes (data not shown). Also no differences in ADMA and SMDA concentrations and arginine:ADMA ratio were observed among the *CPS1* genotypes (table 4).

**Table 4:** Urea cycle intermediates and methylated arginine per genotype.

	CC genotype (n = 71)	CA genotype (n = 43)	AA genotype (n = 14)	*p*
Arginine	41 [24–60]	40 [19–57]	38 [28–60]	NS
Ornithine	87 [60–129]	82 [42–104]	84 [53–114]	NS
Citrulline	21 [17]–[25]	20 [15]–[26]	23 [16]–[26]	NS
Ornithine∶Citrulline	4.1 [3.1–5.5]	4.1 [2.8–5.1]	4.0 [2.6–5.6]	NS
ADMA	0.80 [0.59–1.18]	0.94 [0.59–1.22]	0.84 [0.66–1.35]	NS
SMDA	1.22 [0.94–1.55]	1.19 [0.97–1.53]	1.26 [0.86–1.54]	NS
Arginine∶ADMA	50 [11–70]	40 [26–56]	47 [31–74]	NS

Results of plasma concentrations (µmol/l) are expressed as median [interquartile range].

Statistical analysis between the genotypes. NS = not significant.

## Discussion

Identifying an early biomarker for infants at risk for NEC, i.e. preterm infants, remains an elusive research goal. Both, plasma L-arginine levels [14], [17], [19]–[21] and the T1405N *CPS1* polymorphim [24] have been associated with the development of NEC. When Pearson et al. examined L-arginine and citrulline concentrations in relation to the distribution of the *CPS1* genotypes, they found that term infants who were homozygous (CC) for the C-encoded Thr1405 enzyme had lower L-arginine concentrations than infants with the AA genotype and hence the Asn1405 enzyme [18]. The present study was based on the hypothesis that in a population of preterm infants, in whom the urea cycle is not fully developed, genetically determined variations in CPS1 function would induce further changes in L-arginine levels. However, our data did not confirm this hypothesis because we did not find differences in urea cycle intermediates (i.e. citrulline and arginine) between the different *CPS1* genotypes. This lack of effect of *CPS1* genotype on L-arginine concentrations was also found in adults [22].

L-arginine concentrations as low as 3 µmol/L are sufficient to induce half-maximal activity of endothelial NOS [28], whereas the neonatal L-arginine levels reported in the present and other studies is 10- to 15-fold higher [13], [18], [29]. Clinical and experimental evidence indicates that elevation of the endogenous NOS inhibitor ADMA can cause a relative endothelial L-arginine deficiency, even in the presence of normal plasma L-arginine levels [30]. Elevated plasma levels of ADMA have been reported in diseases related to endothelial dysfunction including peripheral arterial disease, hypertension, hyperlipidemia, diabetes mellitus and hyerhomocysteinemia [15], [31]. However, the information on perinatal ADMA metabolism is limited. There are reports of elevated ADMA concentrations in umbilical venous plasma in neonates born term [32] and elevated levels of ADMA have been involved in the pathogenesis of several neonatal conditions such as persistent pulmonary hypertension or infant respiratory distress syndrome [33], [34]. In a recent study, Richir et al. hypothesized that in addition to low arginine levels, infants with NEC might present increased ADMA plasma concentrations [14]. Surprisingly, a significantly lower ADMA concentration was found in preterm infants with NEC. The levels of ADMA and SDMA and the ratio L-arginine:ADMA that we observed in our study are in the same range as previously reported in preterm infants [14], [34]–[36] and were not affected by the *CPS1* genotype.

Although the present study was not powered to detect differences in amino acid levels or *CPS1* genotype between infants with and without NEC, we observed that the mean L-arginine level of NEC infants showed a non-significant trend to lower values (median 29 µmol/l, interquartile range 16–44 vs. median 40 µmol/l, interquartile range 23–57 in non-NEC group). This trend was not present for citrulline, ADMA or SMDA levels. In addition, we observed that none of the five NEC patients presented the AA genotype. The C-encoded Thr1405 variant of *CPS1* is considered the evolutionarily conserved version, whereas the less frequent, A-encoded Asn1405 variant appears to be a relatively new, gain-of-function mutation [18]. Pearson et al. speculated that individuals with the AA genotype may have an advantage in terms of urea-cycle function and interrelated metabolic processes, especially under conditions of environmental stress [18]. Most typically, NEC has its onset in VLBW infants after the second week of life [37]. One limitation of our study is that we determined L-arginine levels between 6 and 12 hours after birth. It could be speculated that the alterations in arginine and citrulline levels related to genetic variations in CPS1 function may be only present later in life, under the NEC stress conditions, when the demand for NO might suddenly increase. However, it is also possible that the low arginine levels reported in NEC patients [20], [21] could be the consequence, rather than the cause of NEC. Serum citrulline was found in recent years to be a candidate marker of enterocyte mass and intestinal failure [38]. Therefore, in the damaged intestine of NEC infants a lower release of citrulline into the bloodstream may lead to a lower rate of renal arginine production.

Maternal arginine levels may affect the delivery of arginine to the fetus and, consequently, to the infant. One of the limitations in this study is we did not measure amino acid levels in the mother and had no information about the maternal protein intake. Also, we did not measure any index of NO production, such as serum nitrite/nitrate [39]. In addition, our study did not take into account the influence of other enzymes, such as pyrroline-5-carboxylate synthase, argininosuccinate synthase and argininosuccinate lyase, importantly involved in the intestinal arginine-synthetic pathway [17]. However, the activities of these enzymes were shown to be very low, at least in preterm piglets [17]. Interestingly, it has been demonstrated that glucocorticoids stimulated the intestinal expression of pyrroline-5-carboxylate synthase, argininosuccinate synthase and argininosuccinate lyase in neonatal pig enterocytes [40]–[42]. However, we did not observe significant differences in arginine and citrulline levels between patients who received antenatal glucocorticoids (arginine median 40 µmol/l, interquartile range 24–61; citrulline median 21 µmol/l, interquartile range 17–24) and those who did not (arginine median 40 µmol/l, interquartile range 21–57; citrulline median 24 µmol/l, interquartile range 16–30). Finally, our sample size calculation was based upon arginine levels in term newborns [18]. It is important to note that the mean arginine in our patients (AA genotype: 43.5 µmol/L, SD 25.3) was in the range of the one in Pearson's study (AA genotype: 35.7 µmol/L, SD 7.6). However, the larger SD in our patients, reflecting the clinical heterogeneity of preterm infants, means that there might be a possibility of a Type II error underlying our negative results.

In conclusion, the earlier reported association between *CPS1* genotype and L-arginine levels in term infants [18], was not confirmed by the present study in preterm infants. Whether the T1404N *CPS1* polymorphism is a good early biomarker for infants at risk for NEC, needs to be confirmed in a prospective cohort study, which we are conducting at this moment.
